# The Impact of Chinese EFL Teachers’ Emotion Regulation and Resilience on Their Success

**DOI:** 10.3389/fpsyg.2022.898114

**Published:** 2022-05-19

**Authors:** Ling Li, Lili Lv

**Affiliations:** ^1^School of History and Culture, Shandong University, Jinan, China; ^2^School of Translation Studies, Shandong University, Weihai, China

**Keywords:** Chinese EFL teachers, emotion regulation, resilience, teacher success, EFL teachers’ resilience

## Abstract

Success in EFL/ESL contexts by teachers requires developing some essential characteristics consisting of several interacting variables and underlying constructs. With the rise of positive psychology, the concepts of resilience and emotion regulation and their roles in the EFL/ESL milieu have caught attention. In this line, the present study aims to investigate whether there would be any relationship among EFL teachers’ resilience, emotion regulation, and their success in the Chinese context. In addition, we tried to examine the predictive power of EFL teachers’ resilience and emotion regulation toward their success. To that end, an online Likert scale questionnaire including items on teachers’ emotion regulation, teachers’ resilience, and teaching success was distributed to 323 Chinese EFL teachers. The participants were selected based on convenience sampling. Spearman Rho correlation index and multiple regression analysis along with ANOVA were used to analyze the collected data. The findings showed a direct and positive correlation among EFL teachers’ emotion regulation, resilience, and success. Furthermore, it was indicated that EFL teachers’ resilience was more powerful in terms of predicting success than their emotion regulation. Our findings imply that teacher education programs can enhance EFL teachers’ success through training resilience strategies and emotion regulation skills.

## Introduction

Studying teachers’ characteristics as one of the key figures in the educational context and fuel of students’ progress has burgeoned in the educational research. It came to the realization that teachers of English as a foreign language (EFL) can have a tremendous impact on their students’ academic lives and outcomes (Burns and Richards, 2009). Woolfolk and McCune-Nicolich (1984) consider a life-changing role for teachers and argue that most of the teachers’ decisions affect students’ lives. Therefore, studying and keeping a track of teachers’ different psychological and behavioral variables effective in their decision-making process in the classroom and their ultimate success seems worthy and necessary.

In recent years, with the rise of positive psychology and perceiving the importance of teachers’ characteristics, studying emotions experienced by teachers in educational and particularly in second language learning contexts has caught great attention (Nias, 2002; Aragao, 2011; Méndez López and Fabela Cárdenas, 2014; Fathi and Derakhshan, 2019). This increasing attention indicates the fact that besides its role in the quality of teaching, emotion has a great part in both teachers’ and students’ lives (Cross and Hong, 2012). Undoubtedly, due to their various types of interactions with different individuals, such as students and colleagues in their workplace, teachers experience several situations giving rise to emotional involvements affecting their performance (Cowie, 2011). Considering the intensity of language learning situation, teachers and students usually experience several emotions and react distinctively. However, teachers need to manage and regulate their emotions and even sometimes conceal them to achieve their objectives (Sutton, 2004).

Besides the ability to regulate emotions, the capacity of being resilient by teachers earned attention in positive psychology. The term resilience points to an individual’s ability to stand up to adversities and develop appropriate strategies to cope with the situation (Capstick, 2018). Concerning the educational context, teachers need a high level of residency to engage and stay in their work (Williams, 2003). The inevitability of being resilient on the part of both teachers and students to be an impressive player in the stressful situation of teaching and learning is noticeable. In the educational context, teachers need to employ all available resources to maintain productivity and well-being confronting daily difficulties (Day and Gu, 2014). Considering the relationship-based nature of language teaching and learning (Wang and Guan, 2020; Teo et al., 2022), teacher resilience can be flourished in collaboration and contact with students and colleagues, which may lead to teachers’ engagement in their occupation and meaningful actions (Hiver, 2018). Stanford (2001) believes that resilient teachers inherently generate deep involvement and satisfaction in their occupation. Resilience has been studied in association with different educational variables such as work engagement and emotion regulation (Xie, 2021), stress (Rizqi, 2017), and learning motivation (Kim and Kim, 2021). Despite its importance in education, research on resilience is in scarcity (Hiver, 2018), and there is an intense need to study its contribution in instructional contexts.

Achieving success in teaching language, in other words, guiding learners to acquire language successfully, needs EFL teachers to have specific critical capacities, including cognitive, social, and emotional competencies. In addition, they need to be aware of existing challenges and involve with them in the language teaching and learning contexts. Unfortunately, the literature on successful EFL teachers and their key characteristics is inadequate. There are no/few principle-based frameworks concerning the significant features of a successful EFL teacher (Al-Seghayer, 2017).

Considering emotional fluctuation and stressors as principal characteristics of the teaching profession, attention should be given to their manifestation in the classroom. Given the central effects of teachers’ and students’ emotion regulation and resilience on educational success, various studies have been conducted (Jeffrey and Woods, 1996; Lasky, 2000; Bielak and Mystkowsks-Wiertelak, 2020; Wang and Guan, 2020; Fathi et al., 2021; Xie, 2021); However, research on teacher emotion regulation, teacher resilience, and success, particularly in the Chinese EFL context, is in its infancy and demands more attention. Therefore, our study attempts to uncover the correlation between emotion regulation and resilience of EFL teachers and their success and fill the gap by answering the following questions:

1. Is there any correlation among Chinese EFL teachers’ emotion regulation, resilience, and success?
2. Can Chinese EFL teachers’ emotion regulation and resilience predict their success?

## Review of Literature

### Emotion Regulation

With the current interest in positive psychology and consequently increasing attempts to unmask variables affecting L2 teachers and learners, emotion regulation was introduced to the scene of L2 education (Wang et al., 2021). Gross (1998) believes that emotions stem from the intensity and grow within a repeated situation of attention and response. When encountering a problem, an individual starts to attend to it, unfold it, and then initiate a particular emotional reaction. Based on the context, emotions can be constructive and facilitative, for example, when they enhance decision-making, or harmful when they see to “maladaptively biased cognition or behavior.” This is primarily the harmful and destructive emotion that motivates emotion regulation (Gross, 2015).

Since its emergence, it has been defined in different ways. Considering emotion regulation as an intrapersonal function, Gross (1998) defines this term as “processes by which individuals influence which emotions they have, when they have them, and how they experience and express these emotions” (p. 275). Thompson et al. (2008) believe that emotion regulation includes both extrinsic and intrinsic processes bringing about evaluation and managing emotions to accomplish one’s objectives. For Cole et al. (1994, p. 74), emotion regulation is “the ability to respond to the ongoing demands of experience with the range of emotions in a manner that is socially tolerable and sufficiently flexible to permit spontaneous reaction as well as the ability to delay spontaneous reactions as needed.”

Bielak and Mystkowsks-Wiertelak (2020) divide emotion regulation into downregulate and upregulate processes. The former one is employed to decrease and control the effects of negative emotions, while the function of upregulating processes is to increase and intensify the positive emotions. Given that the teaching profession abounds with teacher-student interactions, teachers frequently use emotion regulation strategies. They may employ downregulating strategies to decrease stress or any other negative emotions influencing students’ motivation or level of participation and success. On the other hand, they may benefit from upregulation processes for their emotions to enhance the teaching effectiveness and promote educational achievement (Gong et al., 2013). Similarly, teachers may exploit emotional regulation strategies to generate a supportive relationship with students trying to create an idealized image of an emotional teacher (Sutton, 2010).

Gross (2015) discusses two types of emotion regulation including “intrinsic emotion regulation” and “extrinsic emotion regulation.” Intrinsic regulation of emotions happens when a person, particularly an adult, has the goal of regulating his/her own emotions. In extrinsic regulation, studied in parent–child interactions, a person tries to regulate the emotions of another person. Most of the early works in the area of emotion regulation focused on self-regulation or intrinsic regulation, i.e., intrapersonal perspective (Bielak and Mystkowsks-Wiertelak, 2020), while the recent studies have also called to the scene interpersonal perspectives or extrinsic regulation involving the help of others in emotion regulation (Hofmann, 2014; Barthel et al., 2018). According to Barthel et al. (2018), other regulation of emotions is more effective than self-regulation in that the human brain works with less use of energy when it is supported by others. Other regulation of emotions finds a crucial role in language classrooms since, in the emotionally loaded and sensitive setting of language learning, teachers can provide immense help in handling the emotions of learners (Gkonou and Miller, 2019).

Despite an increasing interest in studying emotion regulation in the various domains of research, few investigations focused on language education, particularly language teacher’s emotion regulation (Talbot and Mercer, 2018; Fathi and Derakhshan, 2019; Bielak and Mystkowsks-Wiertelak, 2020; Greenier et al., 2021). Benesch (2017) argues that in EFL classes, both teachers’ and students’ positive and negative emotions affect the learning outcome, the first one by enhancing and the latter by diminishing learning. Golombek and Doran (2014) believe that given the importance and weight of various interpersonal relationships in the EFL contexts, language teaching is a naturally emotionally demanding profession. Consequently, an inclusive body of research is demanded to understand the significant effect of emotion regulation in the language education domain in various cultures.

Considering the interest in the emotion regulation of ELF teachers, several studies have been conducted in different cultural contexts. Littleton (2021) investigated the strategies used by Japanese EFL teachers in kindergarten to regulate their emotions. Fathi and Derakhshan (2019) studied the Iranian EFL teachers’ stress levels in relation to their emotion regulation capacities. In the Chinese context, few studies have been done on teachers’ emotion regulation, but they also are not in the EFL context. Gong et al. (2013) studied emotions regulation strategies and goals employed by Chinese teachers before and after the class. Their finding showed that Chinese teachers’ aim of regulating emotions was “achieving instructional goals” as well as “decreasing the negative impact of emotions on students learning” (p. 870). In another study, Yin (2016) investigated the process of Chinese teachers’ emotion regulation and its effect on achieving their professional goals. The findings indicated that emotion regulation strategies contribute to teachers’ fulfilling their occupational goals, and consequently may affect their well-being. In line with these studies and to extend the research on emotion regulation, the present work tries to address emotion regulation about Chinese teachers’ resilience and its prediction power concerning EFL teachers’ success.

### Resilience

Initially, the concept of resilience was employed to refer to children’s capacity to adapt themselves to the experienced adversity and grow despite it (Garmezy, 1974 cited in Li et al., 2019). The primary aim of research into resilience in early psychology was to detect distinct personality attributes and other protective factors that can reduce the destructive effects of hardships of life circumstances and lead to positive adaptation (Luthar and Cicchetti, 2000). In the opinion of Patterson et al. (2004, p. 4), resilience, in general, refers to “using energy productively to achieve educational goals in the face of adverse conditions.” Richardson (2002) divides the resilience process into three phases: The first phase relates to the identification of resilience characteristics and qualities such as self-efficacy helping teachers to recover from adversities; the second phase concerns the adaptation process in which the individual tries to develop the resilience qualities. Richardson points to this phase as a “disruptive reintegrative process for accessing resilient qualities” (Richardson, 2002, p.307). In the third phase, a multidisciplinary perspective, the person passes the challenges and is forced by hardships to develop strength over problems.

As a developing field in positive psychology, teacher resilience has been characterized differently. Bobek (2002) defines teacher resilience as a dynamic process reflecting a teacher’s competence to adapt to diverse situations and reinforcing his/her capacity in confrontation with undesired conditions. To Brunetti (2006), teacher resilience is “a quality that enables teachers to maintain their commitment to teaching and teaching practices despite challenging conditions and recurring setbacks” (p. 813). Oswald et al. (2003) consider resilience as an outcome of teacher efficacy and argue that teacher resilience is the ability to get a grip on personal weaknesses and environmental inconveniences, to be able to renew one’s strength confronting potential risks and to keep well-being. Day and Gu (2014) found ethical values and moral courage enhancing factors of teacher resilience and indicated that it is “the capacity to maintain equilibrium and a sense of commitment, agency and moral purpose in the everyday worlds in which teachers teach” (p. 5). Finally, Tait (2008) argues that teacher resilience is relevant to both individual capacity and context, maintains that it is developed in stressful situations through interacting with the environment.

Howard and Johnson (2004), attempting to identify the features of resilient teachers, pointed out that resilient teachers persistently show “a sense of agency, moral purpose, a strong support group, and a sense of accomplishment” (p. 12). In other studies, high morals and positive attitude to their job (Stanford, 2001), ability to manage class and create an effective relationship with students (Day, 2008), and a sense of humor (Bobek, 2002) are found to be the key characteristics of resilient teachers. Bobek (2002, p. 204) pointed out that humor “is vital to strengthening an instructor’s resilience. An instructor who promotes a sense of humor and the ability to laugh at their own errors has an excellent medium for releasing frustrations.” Given the definitions above, Gu and Li (2013) discuss that teacher resilience includes emotional, social-cognitive, and affective components: The first one originates from motivation and commitment; the second is formed from self-efficacy, and job satisfaction is the source of the affective aspect.

Regarding the significant contributions of teacher resilience to positive educational outcomes, some researchers have conducted research on different aspects of teacher resilience (Day and Gu, 2014; Li et al., 2019; Razmjoo and Ayoobiyan, 2019; Stavraki and Karagianni, 2020; Xue, 2021; Wang et al., 2022). For instance, Parsi (2019) inquired about the relationship between the EFL teachers’ resilience and level of creativity in which he found that resilience can predict EFL teachers’ creativity. Stavraki and Karagianni (2020) studied the relationship between the resilience of EFL teachers and their demographic and occupational characteristics. The results showed that demography and occupation did not affect EFL teachers’ resilience, and they suggested that EFL teachers can be trained to enhance their resilience. Xie (2021) examined the prediction strength of Chinese EFL teachers’ emotion regulation and resilience on their engagement in the workplace. The findings indicated both resilience and emotion regulation could moderately predict work engagement. Compared to the quantitative studies of EFL teachers’ resilience, those studies adopting qualitative research methods were relatively rare. However, there were two studies on EFL teachers’ resilience. The first one was conducted by Xue (2021) who adopted a narrative inqury method to self-reflect on her professional development under the umbrella of positive psychology. Another study, which developed resilience by Chinese and Iranian EFL teachers from the cross-cultural perspective, was addressed by Wang et al. (2022). They found that person-focused factors were one of the significant challenges to sustain resilience among EFL teachers. In line with these studies, the present work tries to consider the existence of an association among teacher resilience and the success level of Chinese EFL teachers.

### Teacher Success

Perceiving the contribution of teacher success in education, researchers tried to suggest various models and frameworks concerning the feature of successful teachers (Stronge, 2007). Hung et al. (2007), by defining teacher success as “the sense of achievement which teachers obtain from their work” (p. 415), and by discussing that achievement can include different aspects such as acquiring a skill, effective relationship with students, and getting a promotion. Following the dominant theories of psychology in each era, the effectiveness and success of teachers are interpreted differently. Taking a product-oriented perspective as an example, behaviorism evaluated teacher success in accomplishing predefined behaviors. Cognitivism, believing in a process-oriented approach, weighed teachers based on teaching-learning processes. In the past few decades, the reform movement cast different light upon education, including defining teacher success (Ellett and Teddlie, 2003), whereby various cognitive, social, and affective characteristics incorporated in the evaluation of teachers as effective and successful (Toussi et al., 2011).

Al-Seghayer (2017, p.881) considers five central dimensions, including “cognitive knowledge, content knowledge, English language proficiency, personality traits, key related variables” as the important factors playing a role in an EFL teacher’s success. The personality trait dimension involves flexibility, patience, tolerance, agreeableness, and caring attitude but not issues such as self-esteem, self-efficacy, resilience, or emotion regulation. Campbell et al. (Kyriakides et al., 2002 cited in Toussi et al., 2011) argue that a competent teacher is an expert in the subject matter who is able to structure instructional materials, instruct sufficiently, manage the classroom, exploit instructional time efficiently, supply learners with adequate practice and application of taught subjects, and provide a pleasant environment.

Literature on EFL context shows a growing interest in recognizing factors impacting EFL teachers’ success. The study by Zimmerman and Schunk (2008) indicated that self-regulatory skills affected motivation and achievement, and consequently success. Ghanizadeh and Moafian (2010, 2011) found emotional intelligence and self-efficacy as determinant factors in teachers’ success. In this vein, they investigated the effect of EFL teachers’ critical thinking and found a positive association between critical thinking and success. Examining the contribution of teachers’ stroking behavior in their success, Pishghadam et al. (2021) found a positive correlation between teachers’ positive stroke and their success. Positive stroke could predict success by the mediation of active motivation. Additionally, literature, particularly studies in China, has shown a relationship between EFL teachers’ success and different factors such as their feelings and emotions (Neville, 2013), mastery of teaching techniques (Jie, 1999), self-efficacy, resilience (Tait, 2008), motivation and self-regulation (Fathi et al., 2021), cultural knowledge (Wu, 2017), positive attitude toward research (Derakhshan et al., 2020b), autonomy and professional identity (Derakhshan et al., 2020a), enthusiasm and patience along with the ability to employ teaching methods considering learners’ needs (Huang, 2010), self-reflection, sense of responsibility, and caring for students (Hung et al., 2007).

Overall, considering that teaching is a challenging and complicated task, identifying factors that enhance or impede teacher success seems necessary (Hung et al., 2007). The reviewed evidence in the present study suggests further research on EFL teachers’ success, particularly in the Chinese context in which we could find no work on teacher effectiveness and success. In this line, the present study is going to examine the relationship among Chinese EFL teachers’ resilience, emotion regulation, and their success, along with their predictive power concerning success.

## Methodology

### Participants

The participants consisted of 323 Chinese EFL teachers who were selected through convenience sampling from different universities and colleges across six provinces (Shandong, Zhejiang, Guizhou, Anhui, Fujian, and Hubei) and two municipalities (Beijing and Tianjin). The sample comprises 67 males (20.8%) and 256 females (79.2%), with their ages ranging from 24 to 64. The experience of our participants ranged from 1 to 25 years and more. Participants were also instructed to report their levels of received education and majors. Informed consent was given to all participants. Table 1 presents their detailed demographic information.

**Table 1:** Participants’ demographic information.

Demographic information category	*N*	%
**Gender**		
Male	67	20.8
Female	256	79.2
Total	323	100
**Age**		
24–30	58	17.9
31–40	122	37.8
41–50	111	34.4
51–64	32	9.9
Total	323	100
**Level of education**		
Associate of Arts	81	25
Bachelor of Arts	184	56.9
Master of Arts	19	5.8
Ph.D.	39	12.3
Total	323	100
**Major**		
Applied linguistics	75	23.2
English language literature	60	18.6
English language translation	30	9.2
Teaching English as a foreign language	85	26.5
Teaching English as a second language	33	10.2
Other	40	12.3
Total	323	100
**Teaching experience (year)**		
1–5	81	25
6–10	22	6.8
11–15	57	17.6
16–20	93	28.6
21–25	31	10
More	39	12
Total	323	100

### Instruments

The data for the present study were collected employing a Likert-scale questionnaire, including three separate sections for emotion regulation, resilience, and success. The emotion regulation and resilience scale were adopted from the *Emotion Regulation scale* (Gross and John, 2003), *Resilience Scale* (Campbell-Sills and Stein, 2007), and respectively, teacher success scale prepared by researchers of the present study. The reliability of the questionnaires was estimated by Cronbach Alpha indices and their validity was considered by three experts before conducting the study.

The emotion regulation questionnaire consisted of 10 items with seven points ranging from *strongly disagree* to *strongly agree*. Including 10 items, the teacher resilience Likert scale indicated to what extent the statements are true (not true at all = 0 to true nearly all the time = 4), for them. Finally, the questionnaire of teacher success presented 46 items that measure teachers’ perspectives on their success with five points (1: Strongly disagree to 5: Strongly agree).

### Data Collection Procedure

For conducting the present study, a convenience sampling method was used to collect data from diverse parts of China and different language teaching centers. The questionnaires were distributed online through WeChat *via* Wenjuanxing, an online platform serving for collecting data. All the participants were informed of their rights to withdraw from the study for any reason. They were assured that their identity and personal information would remain anonymous and only be used to meet the aims of this research. After checking the gathered responses for possible outliers, statistical analysis based on Chinese EFL Teachers’ emotion regulation, resilience, and success was conducted.

## Results

### Primary Data Analysis

Before dispersing the questionnaires to the participants, the researchers ran a Cronbach alpha test for each questionnaire to make sure of the reliability of the instruments used for data collection. Table 2 shows that all three questionnaires, including Teacher Emotion Regulation, Teacher Resilience, and Teacher Success, had satisfactory Cronbach alpha indices (0.88, 0.94, and 0.98, respectively).

**Table 2:** Reliability.

Scales	Cronbach’s alpha	Number of items
Emotion regulation	0.884	10
Resilience	0.946	10
Success	0.982	46

After making sure of the reliability of the questionnaires, the researchers ran a normality test to decide whether the data should be analyzed parametrically or not. Table 3 depicts that the collected data were not normal for two of the variables (teacher’ resilience, and success), since the values of *p* for them are 0.000. It is normal only for one of them (emotion regulation). Thus, it violated the assumption of normality, and the data had to be analyzed nonparametrically using a Spearman Rho correlation index.

**Table 3:** Normality test.

	Kolmogorov–Smirnov^a^			Shapiro–Wilk		
	Statistic	*df*	Sig.	Statistic	df	Sig.
Emotion regulation	0.045	323	0.200^*^	0.982	323	0.000
Resilience	0.084	323	0.000	0.977	323	0.000
Success	0.124	323	0.000	0.878	323	0.000

a Lilliefors significance correction.

* This is a lower bound of the true significance.

### The First Research Question

RQ1: Is there any significant relationship among Chinese EFL teachers’ emotion regulation, resilience, and their success?

Spearman Rho index shows the amount and the direction of the relationship among the variables. Table 4 demonstrates that the relationship among the variables is direct (0.585, 0.426), which means that the higher index of one variable, the higher indices of the other variables. Furthermore, the significance level for all of these relationships is 0.000, which means that there is a direct and significant relationship among the variables of the study.

**Table 4:** Correlation among emotion regulation, resilience, and success.

			Emotion regulation	Resilience	Success
Spearman’s rho	Emotion regulation	Correlation coefficient	1.000	0.585^**^	0.426^**^
		Sig. (2-tailed)		0.000	0.000
		*N*	323	323	323
	Resilience	Correlation coefficient	0.585^**^	1.000	0.528^**^
		Sig. (2-tailed)	0.000		0.000
		*N*	323	323	323
	Success	Correlation coefficient	0.426^**^	0.528^**^	1.000
		Sig. (2-tailed)	0.000	0.000	
		*N*	323	323	323

** Correlation is significant at the 0.01 level (2-tailed).

### The Second Research Question

To answer the second research question, which concerns if teachers’ emotion regulation and their resilience in this study significantly predict Chinese EFL teachers’ success, the researcher ran a multiple regression analysis, and the following the model summary in Table 5 was generated.

**Table 5:** Model for the relationship among resilience, emotion regulation, and success.

Model	R	R square	Adjusted R square	Std. error of the estimate
1	0.559^a^	0.31	0.30	20.78

a Predictors: (constant), resilience, emotion regulation.

The model in Table 5 shows the quantity of the variance of the dependent variable scores obtained from the dependent variable (teachers’ success) can be interpreted by the model (which included the teachers’ emotion regulation and their resilience variables). In this model, the value was 0.31 (*R*^2^ = 0.31). Expressing in a percentage, it means that the model (which included scores on teachers’ emotion regulation and their resilience) explained 31% of the variance in scores obtained from teachers’ success.

To estimate the statistical significance of the results, looking at Table 6 labeled ANOVA seems necessary. This examined the hypothesis that multiple R in the population equals zero (0). It met with statistical significance [*F* = (2, 320) = 72.80, Sig = 0.000, which really means *p* < 0.05].

**Table 6:** ANOVA for the relationship among teachers’ resilience, emotion regulation, and success.

Model		Sum of squares	df	Mean square	*F*	Sig.
1	Regression	62913.63	2	31456.81	72.80	0.000^b^
	Residual	138257.78	320	432.05		
	Total	200597.92	322			

a Dependent variable: success.

b Predictors: (constant), resilience, emotion regulation.

To understand which of the variables included in the model can predict the dependent variable more powerfully, the researchers checked the column labeled “Beta” in Table 7. To weigh the differences between variables, it was felt necessary to check the *standardized* coefficients, not the *unstandardized* ones. “Standardized” means that values for each of the different variables being on the same scale so that they can be compared and contrasted.

**Table 7:** Coefficients for teachers’ resilience, emotion regulation, and success.

Model		Unstandardized coefficients		Standardized coefficients	*t*	Sig.
		*B*	Std. error	Beta		
1	(Constant)	122.87	6.80		18.04	0.000
	Emotion regulation	0.42	0.14	0.16	2.96	0.003
	Resilience	1.52	0.19	0.44	7.85	0.000

a Dependent variable: success.

In this study, the researchers were attentive to comparing the contribution of each independent variable; hence, they used the beta values. Looking down the Beta column, they found that the highest beta coefficient was 0.44, which was for teachers’ resilience. This value implies resilience made the most substantial contribution to the existence of the dependent variable at the same time that the variance justified by all other variables in the model was controlled. The other variable’s Beta value (i.e., teaches’ emotion regulation) was also significant since the Sig. value for the group was 0.003, which was less than 0.05.

## Discussion and Conclusion

The leading purpose of this study was to investigate the role of emotion regulation and resilience exercised by Chinese EFL teachers’ in their success. Hence the present study has been done with these objectives: firstly, to investigate the existence of relationship among three variables, i.e., EFL teachers’ resilience, emotion regulation, and success. Then, the predictive function of resilience and emotion regulation concerning success was examined. As to our knowledge from reviewing the literature, the interrelatedness of these three variables has not been investigated among Chinese EFL teachers. Taking into account the lack of extensive research coverage on these novel concepts, we tried to get a line and track the facts instead of supporting the preceding studies.

The results related to research question one indicated a direct positive correlation among teachers’ emotion regulation ability, resilience, and their success. By the way of explanation, ELF teachers who are blessed with higher emotion regulation ability have a higher level of resilience and success and vice versa. Concerning the relationship between teachers’ emotion regulation, resilience, and success, the result was an indication of a significant correlation. These findings confirm Tait (2008), who found that teachers’ personal efficacy, resilience, and emotional competence, including the ability to handle stress and manage emotions, positively correlated with work success. Bobek (2002) argues that resilience and emotional competence are essential factors to EFL teachers’ success, fostered by productive relationships with experienced people, helping them gain new insights into alternatives to deal with different adversities of teaching situations. In the same line, a study by Partovi and Tafazoli (2015) showed that EFL teachers’ resilience correlated with their experience and their success from learners’ point of view. Indeed, our findings corroborate the reported results from Williams, 2003, Wang and Hall (2021), Wang et al. (2022), and Polat and Iskender (2018), all of which, directly or indirectly found a relationship among emotion regulation, resilience, and success. Therefore, supporting the previous studies on resilience and emotion regulation in China (Li et al., 2019; Xie, 2021), the report from the data analysis of the present study indicates that Chinese EFL teachers’ emotion regulation, resilience, and success are tied together.

The direct and positive relationship between teacher emotion regulation and success can be justified by the fact that people who benefit from high levels of emotion regulation can receive more satisfaction from his/her job. Through emotion regulation, teachers manage their emotions and stress when confronting challenges, and consequently are adept in evaluating, adjusting, and directing their emotions, including both positive and negative ones, which leads to satisfaction and enjoyment from their job (Sutton, 2004). On the justification of correlation between resilience and success, it can be said that when teachers learn about the facts and expectations of their work as well as other adversities and stressors, they can develop the necessary strategies by their own experience or by making fruitful relationships with their colleagues and exploiting their experience and knowledge. Therefore, they consider difficulties as challenges and chances to promote not as a threat. Hence, by training to be resilient, teachers empower themselves, maintain their endeavors, and face stressful situations with confidence, trying to alter adversities to opportunities and success (Tait, 2008; Wang et al., 2022).

On the second research question and the predictive power of emotion regulation and resilience concerning success, the findings showed that emotion regulation and resilience could predict success. Considering the direct and positive correlation among these three variables, finding this predictive value seems logical. Since findings to research question one and the previous studies (Tait, 2008; Xie, 2021) showed a reciprocal and positive relationship among the three variables of the study, prediction of work success by the level of emotion regulation and resilience is deductible. Besides understanding the predictive power, we were interested to realize which variable can predict success strongly. The analysis of data indicated that, although both factors can explain the dependent variable significantly, Chinese EFL teachers’ resilience makes a stronger contribution to teachers’ success.

Given the negative correlation between career attrition and teachers’ resilience and their emotional competence (Tait, 2008), the findings of the present study imply that teacher education programs should train resilience strategies and emotion regulation skills and provide resilience-enhancing activities for EFL teachers, particularly the novice ones. They can be exposed to challenging situations through actual classroom observations or videos and encouraged to move directly toward the same situation. In this way, teachers can recognize and enhance their emotional competence and resilience by practicing coping strategies, reviewing one’s position, developing different ways of thinking, working effectively with stakeholders, and other resilient behaviors like self-care and efficacy. On the other hand, EFL education boards should avoid the last-minute hiring of language teachers and provide novice teachers with the opportunity to learn about the adversities of their job, the material they are going to teach to be able to plan and practice. In addition, Wang et al. (2022) recommend teacher educators make EFL teachers broaden their resilience literacy by reading the most recent studies on emotion regulation and teacher resilience, attending teacher-related conferences and workshops, doing action research projects.

Same as other studies, the present study suffers from flaws. The first one was the shadow of the COVID-19 pandemic, which prevented us from reaching the participants in person, and consequently, the questionnaires were filled out online. On the other hand, since the findings are supposed to be used in normal times and refer to them several years from its publication, this temporarily far-working can influence the teachers’ perception of stressors and related concepts and contaminate data. It is suggested that future studies replicate this work in normal times and in-person to avoid the effects of online teaching and online answering to the questionnaires. Second, the present research has generalizing limitations since it has been carried out only in China and in a limited number of cities with EFL teachers. Therefore, applying it to other EFL and ESL contexts should be done with caution. Considering the variables of the present study in both ESL and EFL contexts around the world is advised. In addition, employing a mere closed-ended questionnaire cannot shed light on all aspects of study objects and some points remain unknown. Future studies can include other methods of data collection like open-ended questionnaires, interviews, and retrospectives to have a clearer insight into the relationship between emotion regulation, resilience, and success. Last but not least, we suggest the replication of the present study by the participation of EFL teachers with different years of teaching experience to have their perceptions of success and the effect of their experience on their resilience and emotion regulation.

## Data Availability Statement

The original contributions presented in the study are included in the article/supplementary material, and further inquiries can be directed to the corresponding author.

## Ethics Statement

The studies involving human participants were reviewed and approved by the Shandong University, Weihai Academic Ethics Committee. The patients/participants provided their written informed consent to participate in this study.

## Author Contributions

All authors listed have made a substantial, direct, and intellectual contribution to the work and approved it for publication.

## Conflict of Interest

The authors declare that the research was conducted in the absence of any commercial or financial relationships that could be construed as a potential conflict of interest.

## Publisher’s Note

All claims expressed in this article are solely those of the authors and do not necessarily represent those of their affiliated organizations, or those of the publisher, the editors and the reviewers. Any product that may be evaluated in this article, or claim that may be made by its manufacturer, is not guaranteed or endorsed by the publisher.
